# Liquid-Liquid Phase Separation Promotes Protein Aggregation and Its Implications in Ferroptosis in Parkinson's Disease Dementia

**DOI:** 10.1155/2022/7165387

**Published:** 2022-10-06

**Authors:** Mengzhu Li, Yaohua Fan, Qinglian Li, Xiaoling Wang, Lijun Zhao, Meiling Zhu

**Affiliations:** Shenzhen Hospital of Integrated Traditional Chinese and Western Medicine, Guangzhou University of Chinese Medicine, Shenzhen 518104, China

## Abstract

The pathological features of PDD are represented by dopaminergic neuronal death and intracellular *α*-synuclein (*α*-syn) aggregation. The interaction of iron accumulation with *α*-syn and tau was further explored as an essential pathological mechanism of PDD. However, the links and mechanisms between these factors remain unclear. Studies have shown that the occurrence and development of neurodegenerative diseases such as PDD are closely related to the separation of abnormal phases. Substances such as proteins can form droplets through liquid-liquid phase separation (LLPS) under normal physiological conditions and even undergo further liquid-solid phase transitions to form solid aggregates under disease or regulatory disorders, leading to pathological phenomena. By analyzing the existing literature, we propose that LLPS is the crucial mechanism causing abnormal accumulation of *α*-syn, tau, and other proteins in PDD, and its interaction with iron metabolism disorder is the key factor driving ferroptosis in PDD. Therefore, we believe that LLPS can serve as one of the means to explain the pathological mechanism of PDD. Determining the significance of LLPS in neurodegenerative diseases such as PDD will stimulate interest in research into treatments based on interference with abnormal LLPS.

## 1. Introduction

In the early stage, phase separation or phase transition was applied as a physical and chemical concept to explain the separation of mixed liquids, which was extended as a biological concept to study the occurrence and development of diseases. Several research results present show that phase separation generally exists in proteins, lipids, and other substances and participates in physiological and pathological processes by changing the state of substances [1]. Biological macromolecules can be condensed into membrane-free condensates like liquid through liquid-liquid phase separation (LLPS). Specifically, the viscosity and rigidity of these dynamic droplets increase over time. Through kinetic and thermodynamic ways, they undergo the liquid-solid phase transition to produce a liquid condensate that can mature and solidify, forming a structure similar to amyloid fibrin, and reaching an irreversible and more stable state. Because of the occurrence of many diseases is closely related to the liquid-solid phase transition process of protein and lipid production, such as Parkinson's disease dementia (PDD). LLPS and phase transition (LLPT) of proteins and nucleic acids have become a new research direction in cell biology.

The incidence rate of Parkinson's disease (PD) has gradually increased with age, which has affected about 1% of the elderly over 60 years old [2]. Dementia has become one of the significant public health problems in the world in recent years, with about 7.7 million new cases diagnosed every year, of which PDD accounts for a large part [3]. The characteristic pathology of PDD is amyloid plaque deposition, neurofibrillary tangles (NFT), and massive loss of neurons. These lesions do not evenly wave to all areas of the brain but gradually accumulate to a specific extent showing the neuronal synaptic connection network, namely striatum-substantia nigra, which makes patients have a motor and nonmotor disorders, affecting everyday life and even life safety. So far, it is believed that the etiology and pathogenesis of PDD are related to a combination of factors such as age, aging, genetics, and the environment. However, the pathogenesis of PDD remains unclear. Recent studies have found that phase separation could promote the abnormal aggregation of *α*-synuclein (*α*-syn), an important pathological manifestation of PDD [4]. Thus, we inferred that phase separation might play an essential role in the development of PDD.

Ferroptosis is an iron and lipid peroxide regulatory form of cell death, which has been proved to be widespread in PDD [5]. Studies have shown that *α*-syn and tau are functionally related to iron and lipid metabolism [6–9]. The synergistic action of protein oligomers such as *α*-syn, tau, and iron leads to a vicious cycle of reactive oxygen species (ROS), which further induces lipid peroxidation, causing nerve damage [10–13]. By analyzing the literature, we infer that LLPS affects iron metabolism by regulating protein amyloid aggregation in neurons and liquid-solid phase transition, resulting in ferroptosis and then triggering and exacerbating PDD.

## 2. Mechanism of Phase Separation in Degenerative Diseases

### 2.1. Phase Transitions(LLPT)

Before clarifying the concept of LLPT, we should first know the liquid's definition and characteristics. The liquid is like an “enthusiastic party,” disorganized, easily rearranged, and highly mobile. This is the reason chemical reactions generally occur in liquids. On the contrary, the solid state is more like a “disciplined team,” with internal order and difficulty exchanging components. Like the three-state change of water under factors such as temperature, LLPT refers to the process of matter changing from one phase to another. However, the LLPS phenomenon does not only exist in water. Kato et al. found that biological macromolecules, including proteins and nucleic acids, could be concentrated and self-assembled into protein droplets in vitro using X-ray and other means [14]. Self-assembly of different biomolecular droplets in another liquid (cytoplasm or nucleoplasm) is considered produced by LLPS. Sometimes this physical and chemical process is also called condensation.

### 2.2. Formation and Liquid-Solid Phase Transition of Protein Droplets

Typically, droplet formation in supersaturated solution is a reversible process [15]. The controlling mechanism for forming phase-separated condensates in biological systems arises from multiphase interactions, which can also undergo further transformations [16]. Along with the further study, the researchers found that the average phase separation can be achieved inside cells function area division, assist the cells in the physiological activity, specific protein abnormal phase separation structure tend to be more stable forming more challenging to reverse could become the source of a particular disease, such as neurodegenerative diseases and cancer [17, 18]. For instance, many RNA-binding proteins spontaneously undergo LLP and further condense into amyloid fibrils, forming hydrogels [19]. These amyloid fibrils are usually irreversible or require specific conditions to dissolve, such as high temperature or denaturing agents [20].

In studying biological processes and the pathophysiology of systems, it is essential to understand the principle of molecular condensate formation. It is generally believed that membraneless organelles represented by nucleoli, chromosomes, ribosomes, centrosomes, RNA particles, and stress particles are formed by liquid condensation, so membrane-less organelles are also known as condensate. It has been proved that membraneless organelles play a crucial role in human health, such as dynamic molecular assembly, ribosomal biogenesis, DNA damage response, and intracellular signal transduction [21–23]. Neurodegenerative diseases, including Alzheimer's disease (AD), Amyotrophic lateral sclerosis (ALS), PD, and PDD, are related to the loss of liquid consistency of membraneless organelles. However, the molecular mechanism of membraneless organelles has not been thoroughly studied [24]. Lin et al. speculated that it works through a component-dependent mechanism [25]. Due to the interaction between heterotypic proteins and RNA at the molecular level, when enough biological macromolecules are accumulated in the cell in proportion, the condensate will be formed [26–28]. Although the self-assembly of these proteins follows strict procedures in normal cells, it is unclear how these membraneless organelles maintain their metastable liquid or gel phase to prevent solid aggregation, thereby preventing abnormal phase transformation leading to pathological amyloid aggregation causing neurodegenerative diseases such as PDD [29].

Increasingly evidence showed that LLPS or condensation is the basis for forming membraneless organelles in cells [30]. For example, nucleoli play an important role in ribosomal biogenesis. These nucleolar subsets behave as nonfusible and layered droplet tissues, similar to oil droplets formed in water-rich environments [31]. Because the molecules near the droplet's surface are more unstable than the molecular energy inside the droplet, when two droplets of similar size contact each other, the surface free energy is driven and will spontaneously fuse into larger droplets. This active aggregation was mediated by microtubules in stress granules (SGS), microtubules in the processing body (P-body), and motor proteins [32, 33]. Alberti et al. demonstrated through in vivo imaging and fluorescent labeling of P-body that these particles have liquid properties and can not only fuse, split, and change in size but also reversibly. And the P-body can be continuously exchanged between the external environments or between dilute and condensed phases [34]. These observations require us to rethink how membraneless organelles maintain their shape and composition. Patel et al. concluded that LLPS mediates the formation of membraneless condensates by quantifying cytoplasmic SGS bearing P-bodies in recombinant human cells [35].

Regarding the occurrence mechanism of LLPS, in recent years, the theory of scaffold and client proteins in LLPS has been proposed [36]. The driving molecules of LLPS are called scaffolds, and the client proteins are the proteins involved in the droplets after forming phase separation. The phase separation of the scaffold protein and client protein requires developing an interaction network, often composed of protein-protein interaction and protein-RNA interaction. Proteins characterized by internal disordered regions (IDR) are considered involved in promoting the formation of such interaction networks. Multivalent interaction is an essential factor driving LLPS. LIMD1 as a LIM domain scaffold protein member. The multivalent interaction between IDR and LIM domain synergistically operates LLPS under the regulation of phosphorylation. The LLPS of LIMD1 contributes to cellular mechanics and durotaxis by regulating focal adhesion dynamics in response to force. IDRs within various proteins—including transcription factors, chromatin modulators, and RNA-binding proteins—form liquid droplets via phase separation, which affects myriad biological processes ranging from organelle formation and gene transcription [37]. Transcription factors (TFs), promoter binding site occupancy, and DBD kinetic binding parameters jointly regulate target gene expression [38]. At concentrations greater than critical or saturation (Ccrit), multivalent interactions of proteins and RNA with IDRs drive the formation of phase-separating liquid-like droplets that isolate their constituent components from the surrounding nucleoplasm. The enhancer and promoter drive transcriptional condensate formation to enhance the transcription [39].

Another feature of IDR is that it is composed of a low complexity sequence domain (LCD). For example, FUS, hnRNPA1, and other ALS-related RNP body proteins can form hydrogels composed of amyloid fibrils under conditions such as high temperature, high salt, or high concentration in vitro [40–42]. This is because high concentrations of IDR produced by LLP increase the rate of amyloid fibril formation, leading to droplet maturation [43]. Mutations linked to degenerative disease accelerate this transition [44]. Proteasomal shuttle factor UBQLN2 is an LCD-containing emergency granule protein structurally and functionally different from RNA-binding proteins. The LLPS of UBQLN2 can be affected via Disruption of Multivalent Interactions by ubiquitin [45]. In ALS and frontotemporal dementia (FTD), TDP43-LCD mediates the LLPS of TDP-43, which aggravates the occurrence and development of the disease [46]. Brangwynne et al. demonstrated that phase separation is not the default multivalent interaction mechanism [47]. In recent years, many studies have tried to prove that the use of differential diffusion properties between the condensate and its surrounding cellular environment, as well as across the condensate boundary, is critical evidence of phase separation characterization in quantitative analysis [48]. However, due to the complexity of the biochemical pathways and interactions involved, no definitive conclusions can be drawn [49].

**Figure 1:**
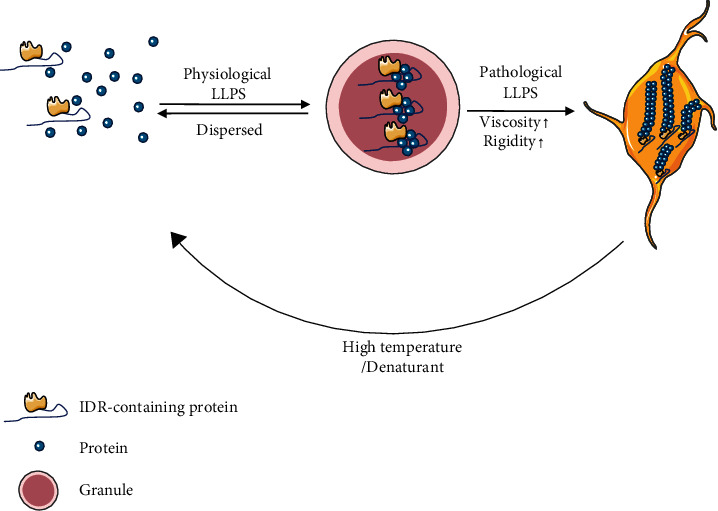
Phase separation and transformation mediate the assembly of protein condensates. The macromolecules, represented by the protein containing IDR, form a reversible condensate during the self-assembly process and then form an irreversible hydrogel form after liquid-solid transformation. These amyloid fibers can be dissolved under specific conditions, such as high temperature and denatured dosage form.

Current studies have shown that condensates exhibiting a liquid metastable state can form solid-like phases through kinetic and thermodynamic pathways, from reversible and dynamic phase transitions to irreversible reactions (liquid to hydrogel to solid-like) over time (Figure 1). Therefore, pathological processes associated with neurodegenerative diseases and aging are also viewed as manifestations of LLP-driven processes [50].

### 2.3. The Role of LLPS in Neurodegenerative Diseases

For many years, the pathological processes leading to aggregation in neurological diseases were thought to be mainly due to the pathological aggregation of proteins prone to misfolding. Neurons, as terminal cells, cannot remove or dilute toxic molecules through mitosis. As a result, they become susceptible to misfolded proteins as they age, leading to further protein accumulation. Because the exposed hydrophobic or abnormal surfaces of the misfolded protein are not recognized by chaperones or ubiquitin ligases, or the rate of formation of the misfolded protein exceeds the rate of chaperone repair and degradation by the ubiquitin-proteasome system, these misfolded proteins will aggregate with each other and lead to protein conformational diseases. Common protein conformation diseases include AD, Huntington's disease (HD), transmissible spongiform encephalopathy, and PDD. With the growing recognition that LLPS may favor protein aggregation, we can now gain a more detailed understanding of the molecular mechanisms underlying the development and progression of age-related diseases [51]. Therefore, pathological processes associated with neurodegenerative diseases and aging are also viewed as manifestations of LLPS-driven processes.

## 3. LLPS Promotes PDD-Related Protein Aggregation

### 3.1. LLPS Promotes *α*-Syn Aggregation

#### 3.1.1. LLPS of *α*-Syn Is Closely Related to Neurodegenerative Diseases Such as PDD

In the genetic study of an early-onset PD family, the role of *α*-syn in PD was revealed by studying its role in DLB patients [52]. *α*-syn is a natural unstructured disordered protein with 140 amino acids, which is divided into three domains: (1) it is mainly responsible for the N-terminal of membrane binding with low complexity and repetition; (2) the hydrophobic central nonamyloidal component region (NAC), which is the core region that forms amyloid fibers, fibrillation, and aggregates into Lewy bodies; and (3) C-terminal with high-negative charge and disorder. These domains combine with cell membrane phospholipids to complete their physiological functions [53]. At present, *α*-syn is highly enriched in the presynaptic membrane and forms amyloid fibers and Lewy bodies through LLPS [54]. Its physiological functions involve various cellular processes, such as maintaining the synaptic function, affecting neurotransmitter storage, and release within synapses. Its aggregation and misfolding to form amyloid formation are thought to be directly related to the pathogenesis of PDD [55].

Aggregation of *α*-syn involves nucleation-dependent aggregation, in which nonstructural proteins first form partially folded intermediates, followed by oligomerization and fibrillation to eventually create amyloid proteins [56–58]. Protein aggregates are highly dynamic and can interconvert on the surface of lipid membranes under specific microenvironmental conditions [59]. Metastable oligomers formed during the early stages of *α*-syn aggregation are toxic in vivo and play a key role in neuronal damage [60] [25]. Some studies have found that abnormally aggregated *α*-syn clumps induce normal *α*-syn molecules to misfold to form polymers by using tiny fibers as “fuzes.” This misfolded *α*-syn is taken up by healthy neurons and acts as templates to replicate within nerve cells causing neurodegenerative changes [61]. Misfolded intermediates gradually form dimers, oligomers, and fibrils by changing pH, temperature, ionic strength, protein concentration, exposure to transition metal ions, exposure to toxins, posttranslational modifications, and other factors [62]. Using a series of well-characterized conformation-sensitive antibodies, Dao et al. found under STED microscopy that protein-formed fibrils release oligomers, which in turn cause fibril spreading-related toxicity that rapidly affects nerve cells, exacerbating the misfolded [63]. Using single-molecule techniques, Garg et al. found that the conformational change of *α*-syn from initially formed oligomers to stable, tighter proteinase K-resistant oligomers is a key step that ultimately leads to fibril formation [64]. The formation of amyloid fibrils not only disrupts the intramolecular interaction between the C-terminus of *α*-syn and the amyloid formation core but also can concentrate the C-terminus on the surface of the fibrils, thus significantly increasing the binding affinity of *α*-syn to the receptor. Phosphorylation of serine 129 (pS129) can further enhance the interaction between *α*-syn fibrils and receptors, leading to pathological *α*-syn aggregation [65].

However, when the protein undergoes LLPS and aggregates into the form of amyloid fibrils, different polymorphs are created, especially in the misfolding aggregation process. The mechanisms involved in this process and its connection with neurodegeneration remain unclear. Experiments confirmed that the *α*-syn phase separated into droplets, which underwent an irreversible transition from liquid to solid, and finally changed to a gel state containing fibrous aggregates and oligomers [66]. The formation mechanism of amyloid fibrils is the formation of core structures mediated by sequence-specific interactions between *α*-syn monomers. Mutation experiments suggest that intramolecular interactions, such as electrostatic and hydrophobic, may exist between the N-terminal and the NAC domains of *α*-syn. It showed that an LCD at the N-terminal and central hydrophobic NAC domain is the primary driver of *α*-syn LLPS, and the hydrophobic plaques in the *α*-syn core region are critical sites for LLPS and amyloid aggregation. Zhang et al. revealed that the C-terminus could improve cytotoxicity and *α*-syn aggregation by interfering with N-terminus binding to membrane and molecular chaperones [67]. The C-terminal domain can regulate *α*-syn phase separation through electrostatic interactions. Truncated *α*-syn accelerates the conversion of W.T. *α*-syn into amyloid aggregates by regulating phase separation [68]. It has been shown that the presence of salt/counterion concentration, pH, presence of surface, PD-associated multivalent cations, and N-terminal acetylation promotes hydrophobic interactions of LLPS, resulting in charge neutralization of the two terminal parts of the protein. Therefore, regulating salt content can induce *α*-syn to undergo spontaneous (transient) or delayed LLPS in vitro [69]. At the same time, the study showed that *α*-syn could generate LLPS in the presence of PEG, suggesting that PEG can induce LLPS through increased local concentration and protein binding through induced molecular crowding [70]. Past research believed that *α*-syn could form a relatively stable state by folding. Dopamine can disrupt the long-range molecular interactions between the C-terminus and NAC, breaking the stable, compact conformation, causing fibrillation, and promoting amyloid formation.

When the aged droplets transform into a gel-like state, the monomer content decreases, which benefits the increase of fibers. In amyloidosis, fibrillar aggregates deposit in neuronal cells through the formation of intracellular inclusion bodies or amyloid extracellular plaques, triggering neuronal degeneration, which leads to manifestations of various neurodegenerative diseases. *α*-syn has an extreme polymorphism after aggregation through LLPS. Mutations at different disease sites can induce the formation of different structures, resulting in new toxicity and properties. The misfolding and abnormal aggregation of *α*-syn undergoing LLPS may be a toxic process associated with PDD.

#### 3.1.2. The Factors which Promote PDD Accelerate *α*-Syn Aggregation and Droplet Formation

PDD is a sporadic disease. In addition to the environmental and cellular factors that play a significant role, metal ions, interaction with the lipid membrane, posttranslational modification of proteins, and familial mutations also significantly impact the concentration of *α*-syn in the diseased brain and in vitro [71]. When there are regulators (Cu^2+^, Fe^3+^, Mn ^2+^, and liposomes) in droplet formation, researchers observed faster droplet formation [72]. In addition, lipid and other factors also reduce the critical concentration of *α*-syn in LLPS [66]. These related factors promote LLPS by reducing the solubility limit of *α*-syn and increasing the internal hardness of droplets. Studies have shown that regulating factors affecting protein assembly or solubility, such as posttranslational modification and pH values, play a crucial role in regulating LLPS [73]. In conclusion, the phase separation of *α*-syn is related to its aggregation behavior in the presence of PDD-related factors.

### 3.2. The Aggregated Tau Has a Strong LLPS Tendency

Tau is a disordered and highly soluble microtubule-associated protein released from the full-length protein through protease and endopeptidases and participates in tubulin assembly and axon transport [74, 75]. At present, the mechanism of inducing its transformation from nontoxic tau monomer form to solid-like form with neurotoxin deposition is still unknown.

Because the number of insertions at the N-terminal differs, the tau subtype forms an incompletely repetitive sequence (R1-R4). Tau produces six tau isomers by alternative splicing, and the N1 variant of the repetitive sequence of the N-terminal is the most expressed in the human brain [76, 77]. Current studies have found that tau aggregation is closely related to Down syndrome, pick's a disease, progressive supranuclear palsy (PSP), FTD, PDD, and so on [78]. Among them, up to 50% of PDD patients have a large number of NFTs containing tau. One of the characteristics is the misfolding and pathological accumulation of microtubule-associated protein [79]. At the same time, it may be related to pathological aggregation *α*-syn accelerates the disease process and leads to a worse prognosis [80].

There are many reasons for tau fibrillation, such as viscosity and phosphorylation modification. Microtubule binding repeats of tau rich in lysine have a strong tendency for LLPS in solution, producing droplets representing the supersaturated metastable state of protein [81]. Due to the significant increase in protein concentration, tau 441 fibrillation accelerated sharply under LLPS conditions. At the same time, it is speculated that the droplet environment may change the conformation of tau protein, making it more prone to fibrillation. On the other hand, droplets with increased viscosity may slow down the fibrillation. Experiments show that when two tau isomers with different fibrillation tendencies are aggregated into droplets, the other tau subtype with a lower aggregation tendency may significantly reduce the fibrillation rate of rapidly aggregated species, which is unique to LLPS [82]. Besides, there is evidence that posttranslational modification can regulate the LLPS of tau, such as the promotion of phosphorylase. Luk et al. proved that tau repeats could be decomposed into droplets by LLPS under posttranslational modification, especially phosphorylation. It is proposed that the droplets formed by tau's positively charged microtubule-binding domain agglomerate with negatively charged molecules to promote the formation of amyloid [83]. Due to the small number of hydrophobic residues and the low complexity of their amino acid sequences, tau has almost no aggregation trend in vivo [84]. Some experiments have found that the binding of negative charge factor and tau is not enough to trigger the formation of tau amyloid. LLPS can lead to the molecular crowding of tau amyloid-promoting elements and drive electrostatic condensation. Cremades et al. and Wen et al. showed by truncation experiment that in addition to electrostatic driven LLPS, tau protein could also cause LLPS through hydrophobicity mediated by its C-terminal domain [85, 86]. It has been shown that the IDR of tau can drive LLPS through the network of weak multivalent interactions between short motifs via transient or reversible physical contacts [87].

Zhang et al. proposed that droplets formed by tau's positively charged microtubule-binding domain agglomerate with negatively charged molecules to promote the formation of amyloid [88]. These tau reaction chambers with high positive charge density established by liquid stratification can recruit polyanion aggregation promoters. Various molecules have been proven to promote the fibrosis of tau and the aggregation of amyloid into NFTs through liquid-solid phase transformation. Among them, polyanionic factors such as heparin are particularly effective, but at the same time, specific but unknown phosphorylation modes are required [64, 89]. LLPS without heparin and heparin-induced fibrosis depend on the temperature and ionic strength of K18 solution similarly, while heparin-induced K18 fibrosis can form amyloid more efficiently at alkaline pH [73, 90]. Recent work also showed that following incubation with PEG, the prolonged LLPS of tau leads to the adoption of known pathogenic conformations of tau, such as oligomers and N-terminal exposure. However, the neurotoxicity caused by LLPS has not been evaluated [91]. The interaction of tau with TiA1 in SGS can accelerate the formation of tau LLPS and toxic oligomers [92].

Therefore, it can be concluded that LLPS can promote the misfolding and abnormal accumulation of tau protein and aggravate fibrillation. At the same time, when the reduction conditions and physiological temperature remain unchanged, increasing the local concentration of tau repeats and supplementing polyanions such as heparin in the process of LLPS can promote the formation of phase separation droplets and make tau proteins aggregate and fold into amyloid fibers, to further accelerate the process of disease.

### 3.3. LLPS Plays an Essential Role in the Pathological Coaggregation of *α*-Syn and Tau

*α*-syn and tau play important roles in the development and maintenance of normal function of the nervous system. Pathological *α*-syn and tau mainly aggregate in the brain stem, cingulate gyrus, and entorhinal cortex. Different neurodegenerative diseases have other protein precipitation locations [93]. However, there are still many overlapping clinical features, such as *α*-syn and tau protein copolymers in some PDD patients [94]. Some studies have proved that *α*-syn and tau can occur in both self-aggregation and coaggregation in vivo and in vitro. Studies have found that Tau colocalizes in *α*-syn-rich disease inclusion bodies and is separated into liquid condensates by electrostatic complex coacervation, and the condensates formed undergo either fast gelation or coalescence and eventually form amyloid aggregation [95]. Compared with *α*-syn of small molecular weight and high concentration that can occur in vivo, tau self-aggregation requires the participation of cofactors (such as aminoglycan or nucleic acid) [96]. Tau protein is readily phosphorylated due to its high content of Ser and Thr. It is known that tau protein contains 85 potential phosphorylation sites, mainly located at the C-terminal of the proline-rich region and adjacent microtubule binding region. The C-terminal domain of *α*-syn binds to the proline-rich P2 region of Tau; glycogen synthase kinase 3*β* promotes Tau phosphorylation by interacting with protein kinase A (PKA) and glycogen synthase kinase 3*β*, which is essential for tau to promote the assembly of tubulin into microtubules [97]. This process can be inhibited by Cdk2 phosphorylation [98]. The C-terminal of *α*-syn can directly bind to the motif of the microtubule-binding domain of tau. They interact with each other through the PHF6 motif to synergistically promote coaggregation. Ambadipudi et al. artificially excluded the secondary effect and used purified compounds to prove that *α*-syn induced tau fibrosis and that coaggregation of tau and *α*-syn synergistically promoted the oligomerization and fibrosis of the two proteins. To exclude secondary effects, Ambadipudi et al. demonstrated that *α*-syn induced tau fibrosis using purified compounds and that coaggregation of tau and *α*-syn synergistically promoted oligomerization and fibrosis of the two proteins [99].

**Figure 2:**
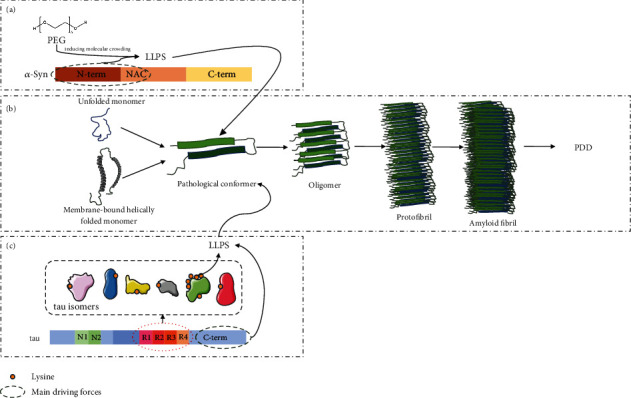
Molecular mechanisms of PDD-related protein aggregation involved in LLPS. LLPS drives abnormal aggregation and denaturation of *α*-syn and tau. (a) Driven by the N-terminal and NAC region, *α*-syn can combine with PEG to produce the LLPS process. (b) The unfolded biomer and membrane-bound helically folded biomer can be combined into a pathological conformer, undergo an irreversible transition from liquid to solid, and finally change to a gel state containing fibrous aggregates and oligomers, ultimately leading to PDD (c). In addition to C-terminal mediated and driven LLPS, based on the number of insertions at the N-terminal is different, the tau subtype forms an incompletely repetitive sequence (R1-R4), which has a strong LLPS tendency.

Monahan et al. found in the study of the Drosophila PDD model that the interaction between *α*-syn and tau can enhance neurotoxicity [100]. The formation of early heterotypic oligomeric complexes of *α*-syn and tau K18 can promote fibril maturation through binding-induced misfolding and aggregation. Heterotypic amyloid and its intermediates may exhibit higher toxicity than the homooligomer intermediates [101]. The *α*-syn-dependent C-terminal domain is easily and directly bound to the proline-rich P2 region of tau protein and is regulated by tau phosphorylation. Full-length *α*-syn, rather than the carboxyl terminus truncated, is concentrated in tau droplets. Tau proteins and their different parts are prone to phase transitions. Besides, the repeat region undergoes LLPS at pH values close to its photoluminescence (PL) [102]. To sum up, it is of great significance to explore how to use LLPS theory to analyze and cut off the pathological coaggregation of *α*-syn and tau, which can reduce cytotoxicity and delay the occurrence and development of neurodegenerative diseases such as PDD (Figure 2).

## 4. The Significance of LLPS-Induced Protein Aggregation Caused Ferroptosis

Iron is the most abundant transition metal in the brain and is essential for various neuronal functions. The imbalance of iron homeostasis is inextricably related to the mechanism of PDD. Ferroptosis is a kind of cell death different from apoptosis and other ways of iron-dependent regulatory forms of cell death. At the end of containing polyunsaturated fatty acyl of phospholipids, oxidation occurs in an iron-dependent way, cellular mitochondria swelling, morphological changes, and the characteristics of the accumulation of lipid peroxides [103]. It has been shown that ferroptosis are widespread in the PDD in vitro and in vivo models [104], and lipophilic antioxidants and iron chelating agents inhibit ferroptosis [105, 106]. The traditional pathological features of PDD include the death of dopaminergic neurons in the substantia nigra and the deposition of intracellular proteins such as *α*-syn. In addition, iron accumulation elevated oxidative stress, and lipid peroxidation is related to neurodegeneration through unknown mechanisms. The high iron phenomenon observed in the substantia nigra of PD subjects is considered due to the lack of regulation of ferritin and transferrin or the presence of nitric oxide, which promotes the entry of iron into dopaminergic neurons, thus, stimulating oxidative stress and protein changes, including *α*-syn aggregation and cell death, resulting in neuronal damage [107, 108]. Under the pathological conditions of free unstable iron and elevated oxidative stress, *α*-syn directly or indirectly enriches the cell membrane of the amino acid (AA) and other polyunsaturated fatty acids (PUFA), which may lead to further lipid peroxidation and promote ferroptosis of neurons [109–111]. Avila et al. demonstrated that ferroptosis driven by *α*-syn oligomer binding plasma membrane is closely related to lipid peroxidation by establishing a human stem cell-derived model of synuclein disease characterized by *α*-syn and proposed ferroptosis as the pathological mechanism of synuclein disease [112]. Therefore, analyzing the role of iron metabolism and lipid peroxidation in neuronal death and clarifying the molecular mechanism of ferroptosis and oxidative stress-induced neurodegeneration are the key steps to deciphering the pathology of PDD.

### 4.1. Iron Catalyzes LLPS of *α*-Syn

Early studies found that cells exposed to dopamine alone did not cause aggregation of *α*-syn, but iron-treated cells did induce aggregation. Treatment of cells with metal ions (such as Fe^3+^) can induce ROS formation and promote the aggregation of *α*-syn in cells overexpressing proteins [113, 114]. Fe^2+^ and Fe^3+^ strongly bind to *α*-syn and convert this internally disordered protein into a *β*-Folding structure to promote its oligomerization [115]. Data suggests that diseases caused by increasing intracellular concentration, such as a dose effect of the gene, autophagy injury, or proteasome dysfunction, may promote the accumulation of iron in neurons and then further promote the oligomerization and aggregation of *α*-syn [116–118]. Panza et al. found that the presence of *α*-syn aggregates alone did not affect cell viability during the gradual transformation of droplets into solid-like aggregates. In contrast, iron-induced *α*-syn aggregation can lead to neurotoxicity of neuronal cells [119]. By monitoring the colocalization of *α*-syn and Proteostat and calculating the attack tendency factor at different times after iron treatment, it is suggested that iron-induced LLPS can trigger the formation and proliferation of *α*-syn invasion. Still, the mechanism is not clear at present [120].

### 4.2. *α*-Syn Plays a Vital Role in Iron Homeostasis

Iron is a trace element in the form of Fe^2+^ or Fe^3+^ in vivo. As a cofactor of the enzyme, it is isolated from ferritin. Generally, iron toxicity is mainly driven by Fenton and Haber-Weiss reactions. During neurodegenerative diseases such as PDD, the brain iron level can be detected in living tissue and postmortem tissue [121]. Many studies and reviews have explored the interaction between *α*-syn and iron metabolism, which has found that *α*-syn plays a vital role in maintaining iron homeostasis [122].

The posttranslational modification of *α*-syn can regulate iron transport. Acetylation of *α*-syn at the N-terminal promotes dynamic protein-mediated transferrin receptor (TfR) endosomal transport and iron internalization. Siegert et al. suggested that *α*-syn directly mediates iron metabolism by promoting transferrin-bound iron uptake and colocates with transferrin receptor 1 (TfR1) in the plasma membrane. The absence of *α*-syn causes TfR to become trapped in circulating endosomes, depleting the cell's iron reserves [123]. At the same time, iron exposure to neuronal cultures overexpressing *α*-syn with familial mutation (A53T *α*-syn) increases the formation of aggregates and susceptibility to iron-induced toxicity [124]. Phosphorylated *α*-syn can reduce iron input through TfR endocytosis [125]. In neurons exposed to excess iron, overexpression of *α*-syn leads to increased intracellular iron levels and redistribution of iron from the cytoplasm to the perinuclear region in *α*-syn-rich inclusions [126].

*α*-syn regulates iron content. As an iron-reductase and iron-binding protein, *α*-syn reduces Fe^3+^ to Fe^2+^, and the accumulation of *α*-syn increases the amount of iron in cells, thereby increasing oxidative toxicity and sensitivity to the production of iron-dependent reactive oxygen species (ROS) and lipid peroxides (LOOH) [61]. Finally, oxidative stress and neuronal degeneration were induced by the Fenton reaction. The increase of *α*-syn can affect lysosomal activity by interrupting the transport of lysosomal hydrolase, damaging ferritin phage, and participating in the process related to ferroptosis [127].

The results of Martin et al. showed that iron deposition could occur in the monkey substantia nigra-striatum system after intranasal injection of exogenous *α*-syn preformed fibrils (*α*-syn-PFFs). It is suggested that the potential mechanism may be that *α*-syn-PFFs treatment can trigger the accumulation of de iron in microglia to start the neuroinflammatory response and then induce the cascade reaction between iron deposition and microglia activation, produce hydrogen peroxide and hydroxyl free radicals, release proinflammatory factors, and trigger neuroinflammation *α*-syn aggregation and degeneration of dopaminergic neurons [128]. *α*-syn contains an iron response element (IRE) in its 5'UTR mRNA region, which regulates the binding site of protein translation when regulating the iron load of neurons [129]. As an iron regulatory protein, iron deficiency leading to *α*-syn translation decreased, while overexpression of *α*-syn in neurons resulted in higher levels of Fe^2+^ [130]. Intracellular processing of amyloid may impair iron export by destroying the stability of iron transporters on the cell surface. On the contrary, the nonamyloid processing of amyloid precursor protein (APP) on the cell surface promotes iron transporters' stability, reducing the amount of iron in neurons [131]. Therefore, iron metabolism disturbance induced by *α*-syn is an important factor in ferroptosis.

### 4.3. Tau Experiencing LLPS Affects the Iron Deposition

In the case of molecular crowing, tau can experience LLPS and concentrate the protein to form highly dynamic droplets. Tau can interact with protein monomers in vitro and in vivo to form amyloid fibers, ribbon fibers, and/or straight filaments (SFS) [132]. Elevated iron levels were found in brain regions that accumulate NFT with paired helical filaments (PHFs) [133]. This is because the ability of iron can lead to binding to PHF and can regulate tau phosphorylation or even induce tau hyperphosphorylation by inducing the activity of a variety of kinases. Iron also induces hyperphosphorylated tau aggregation and promotes the formation of NFTs through the direct interaction of the putative iron binding motif in tau protein. NFTs formed by misfolding and aggregation are widely found in PDD. The abnormal accumulation of tau can lead to iron deposition, which relies on iron-induced oxidative damage to produce toxicity, resulting in neuron loss or ferroptosis [134]. APP can interact with iron transporters on the surface of neurons to regulate iron output. The loss of tau leads to the accumulation of immature app in the endoplasmic reticulum, prevents the app from being transported to the surface of neurons, and then leads to the toxic accumulation of iron [135].

**Figure 3:**
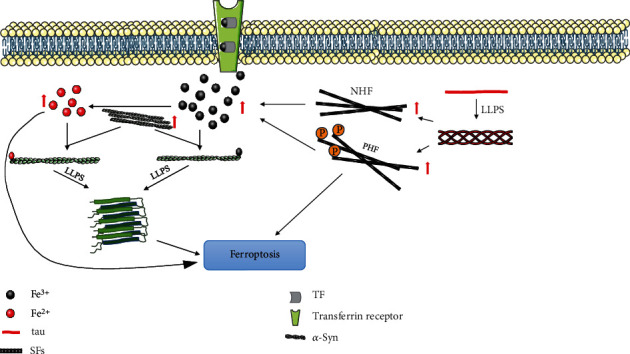
Mechanism of LLPS affecting ferroptosis. (a) Iron ion induces LLPs process of *α*-syn, *α*-syn can promote the uptake of iron ions by transferrin and regulate iron transport. The abnormal aggregation of *α*-syn can reduce Fe^3+^ to Fe^2+^, resulting in the increase of iron content, the disorder of iron metabolism, and ferroptosis. (b) Tau posttranslational modification represented by phosphorylation promotes the formation of NFT, causes oxidative damage by affecting iron deposition, and finally leads to ferroptosis.

Because the environment in the LLPS droplet is different from that in the surrounding aqueous phase, LLPS significantly impacts the formation of pathological protein aggregation in dementia and other neurodegenerative diseases, and the abnormally accumulated protein plays a role in ferroptosis [136]. The occurrence of PDD pathology is closely related to iron metabolism. Applying the principle of LLPS to explore the relationship between tau, *α*-syn, and iron is of great positive significance to further understanding the pathological mechanism of PDD. (Figure 3).

## 5. Discussion

As an essential nonmotor symptom of PD, PDD is characterized by loss, degeneration, or necrosis of dopaminergic neurons in the substantia nigra, significantly reduced dopamine content in the striatum, and misfolding of *α*-syn and other proteins. Its etiology and pathogenesis are unknown, so there is no effective prevention and cure measure. Literature research shows that LLPS is the crucial mechanism of abnormal accumulation of *α*-syn, tau, and other proteins in PDD. Based on the latest research, they inferred that LLPS could indeed be used as a method to explain the pathological mechanism of PDD. This method will help determine the most suitable node for treatment targeting to develop new treatment strategies.

When the pathological process starts, *α*-syn, tau, and other specific proteins are mixed with other molecules and separated from the cytoplasm to form small droplets. The viscosity of these tiny droplets will gradually become stronger and eventually become very hard. During this process, the pathological accumulation of *α*-syn and tau simultaneously affects iron metabolism and causes iron deposition. After the iron homeostasis is broken, a significant increase in iron deposition triggers ROS accumulation. Eventually, it leads to neuronal ferroptosis, contributing to the vulnerability of dopaminergic neurons or even to a vicious cycle of toxicity in the pathology of PDD. Therefore, we believe that the ferroptosis process can be reduced or delayed by intervening in LLPS of *α*-syn and tau so as to improve the incidence of PDD.

As a physical and chemical phenomenon in the early stage, LLPS is now extended to the biomedical field. As a new research method, it is used to explore the pathological mechanism caused by the liquefaction and coagulation of proteins, lipids, and other substances and to explore the treatment methods for cancer, neurodegenerative diseases, inflammation, and other diseases. Everything has two sides. The vigorous development inevitably leads to a certain degree of abuse, which is easily confused with other pathological phenomena in the research. It is suggested that we should pay close attention to the changes in molecular physical and chemical properties in the process of the experiment to avoid drawing wrong conclusions.

Currently, the research and clinical evidence on the etiology and pathogenesis of PDD are still minimal and inconclusive. Therefore, clarifying the occurrence process and the principle of neurodegeneration and looking for dopaminergic neuron compensation methods are of great significance in promoting the recovery of neural function in patients with PDD. LLPS belongs to a physical and chemical phenomenon across multiple fields. Therefore, combining multiple disciplines to carry out horizontal and vertical research on the possible mechanism of cognitive impairment in PD will help to early identify high-risk groups with cognitive impairment, cut off the disease's development path and delay PDD's progress.
